# Our House and Our Servant

**Published:** 1903-07

**Authors:** H. McHatton

**Affiliations:** Macon, Ga.


### OUR HOUSE AND OUR SERVANT.*

BY H. McHATTON, M.D.,

MACON, GA.

One of the most important questions before our profession today is preventive medicine. It may not give one as much glory to prevent a case of typhoid fever as it does to treat one, but in my estimation it gives him more personal satisfaction.

The vast importance of this suhject can only be realized by us who know that at least fifty per cent. of all deaths are caused by diseases that are absolutely preventable. We also know that our State is far behind the times in this work, and that it is only by

*Read at the Columbus meeting of the Medical Association of Georgia.

constant ventilation of the subject that we can ever hope to get the cooperation of our officials. In the meanwhile we should spare no effort to educate our patients up to the proper appreciation of the easily avoidable dangers that they run daily, and in this way save numberless valuable lives each year.

In the time allotted to me, it is impossible to go into this subject in its entirety, so I shall only take up some of the ordinary risks connected with our daily life.

Our house—referring only to the house we move into as a purchaser, tenant or boarder—not to the house that we build.

When a man contracts to buy a house, the first thing he does is to consult a lawyer as to the validity of the title. Does it ever occur to him to consult a doctor as to its medical history? No—he sees no reason to do so; and in the majority of instances, in my experience, where this information has been volunteered, it has not been appreciated or even pleasantly received.

I do not hesitate to say that there is not a gentleman present who has practiced his profession in one locality for ten years who does not know more houses with bad medical histories than with defective titles, or who could not point out a number of houses that he would not take as a gift provided he had to move into them as they stand and live there one year. We know them, that they are infected, and that the danger of living in them is greater than that of a soldier going into battle. Still, as a rule, we allow our patients to move into them with at most a mild protest. Why? Principally because our advice is not asked, and voluntary advice along these lines is rarely followed. Most of these houses can be rendered perfectly safe at a moderate cost; some cannot at any price, because we are unable to find the source of infection.

We will leave out of discussion the well-known contagious diseases, such as scarlet fever and smallpox, for at present, in most of these cases, there is some slight effort at disinfection; but take tuberculosis—until recently the most deadly disease in our mortuary statistics. This is not the place to discuss its etiology. Sufficeth to say that there is probably not a single authority in our scientific world who would deny that in the vast majority of cases it is acquired by taking into our system the tubercle bacillus.

Personally I am of opinion that in ten years the physician that considers this disease as purely hereditary will be on the plane of the surgeon of to-day that does not believe in antisepsis or asepsis.

We do not know how many of these bacilli it would take to infect a properly prepared subject; neither do we know how long a given bacillus preserves its infecting power in proper environment. Certainly for many months, and probably for many years. We have them to spare, so we need not figure too close. When we remember that the consumptive with a cavity expectorates from two billion five hundred million to four billion bacilli each twenty-four hours (Nuttall), we can form some idea of the condition of a house that has been occupied by one or more of these unfortunates for years with no practical effort at sanitation.

I have in my mind a house of moderate size which has had from one to three tuberculous inhabitants all the time for the past ten years. The house is now on the market.

A few years ago a tuberculous patient moved into a room in a boarding-house of the better class in an adjoining city and died. The room was taken by the next applicant, who died in due time, followed by the second with the same fate and by the third who, when last heard from, was in the last stages of the disease.

Some time since I saw in consultation a child with tubercular meningitis, and incidentally its father with pulmonary tuberculosis. They both died and were followed in the house by a young married couple from the country. The woman was a magnificent physical specimen with a perfect family history. Within a comparatively short time I saw her in consultation—tubercular meningitis.

An infected house was vacated for a few months by its owners. A family of three, all well and of good family and personal histories, moved in. One of these died in less than eighteen months and a transient visitor became infected.

In 1895 I took charge of an institution in my city, the occupants, adults, varying in number from twenty to twenty-five. In the previous ten years there had been ten deaths, one each year, all from tuberculosis. In the past seven years there has not been a death from this disease.

The following history is taken from that valuable work of Latham on Tuberculosis: "A newly-built flat in a fairly sanitary condition, but badly lighted and ventilated, had been occupied for eight years by three families in succession; all of them had presented a clean list of health until the family X took up their residence in the same quarters. In this family the mother was consumptive when she came. She died in the flat. Shortly afterwards the family left, having lived there for one year only. The flat was next occupied by the family Y of seven persons, all healthy. After a year's stay they left, and some years later the father, mother and one son died of phthisis, and a boy of chronic peritonitis. A third family, Z, all healthy to begin with, next took the rooms. One child died of meningitis, another of marasmus, and a third contracted hip disease; subsequently the father died of phthisis, another child of meningitis, the mother acquired consumption, and a child became scrofulous. A fourth healthy family, W, next came into residence. After a time the mother became phthisical, and two children died of meningitis. During the whole period over which these observations range the flat was never empty and was never painted or cleaned. In other parts of the same building, which were properly cleaned, no case of tuberculosis occurred.

We may take up the same line of argument on typhoid fever. I will give only one example.

In the fall of 1895 a religious institution came under my professional charge, with the following history of typhoid fever: In 1890, five cases; 1891, six; 1892, three; 1893, ten; 1894, four; 1895, twelve.

This institution has an average population of fifty. Their medical attendant's orders are implicitly followed, regardless of cost.

Since 1895 there has been one case, and that unquestionably contracted in a camp during the last war.

We have another class of infected houses where our best efforts are of no avail in getting a satisfactory explanation of the cause. As an example I may cite the following:

A large, well-built house on one of the highest hills in my city became infected with malaria of the gravest type. Three families moved in in succession, and each of them moved out in less than a year on account of this infection. Not less than fifty per cent. of the inhabitants of this house were victims to the severer types of malaria during the time it was under my observation, a period of about three years. There were three other houses within a radius of fifty yards, and in none of them was there a case of malaria during this time. It is needless to say that every supposed cause of malaria was investigated with negative results. This house was becoming a veritable nightmare to me when fortunately it was destroyed by fire.

OUR SERVANT.

The servant question at present is in a transition stage, and becoming more serious each day.

Prior to 1861, on account of the constant personal supervision, medical attention, curbing of natural vices and gregarious tendencies, with general hygienic surroundings, our servant was as nearly perfect physically as any human being could be.

What they were personally can best be shown by the absolute affection that we all have for our old mammies and uncles, and the numbers of them that are pensioners, after forty years, of the children that they nursed.

The servant of to-day is another being. They are deteriorating so rapidly that only the country negro keeps up the race.

As a class they have no supervision, are addicted to the worst of vices and live in misery in the vilest of hygienic surroundings. Even the better class do not get the medical attention and good environment of the old days, and their gregarious tendencies subject them to exposure to all prevalent contagious diseases.

They leave us at night and report in the morning, sometimes. What do we know or care how the intervening time is passed?

Our child's nurse often has spent the night in the same room with a case of measles, diphtheria, smallpox or tuberculosis, of which fact we may eventually become aware if the case dies.

And our wife's dude butler. Suppose she even had the faintest idea of his progress of the previous night through the negro tenderloin of any of our cities. A vast majority of our epidemic diseases are brought into our families through our servants. To-day smallpox is epidemic in the State. In the cities at least ninety per cent. of it is among the negroes. How many of us know whether our servant is vaccinated or not. But there are other diseases. Think of the pitiable spectacle of the young mother leaving her home to save her first born from the possible contagion of the case of scarlet fever around the corner, or the case of measles across the street, and said first born making the trip in the arms of a nurse with tuberculosis or a mouth full of mucous patches.

In answer to an advertisement for a wet nurse not long since, I found three awaiting my arrival at the office—two tubercular. I had in an old ante-bellum aunty a pet patient whom I had been carrying for some time as best I could, with an advancing tuberculosis. She was a cook. Not reporting, I made inquiries about her and found that she was so sick that she had gone to nursing and was sleeping in the room with two children. We all know the negro's idea of ventilation, especially when they have a bad cold.

I had a rather fashionable monthly nurse some years since who infected six in one family for me, and several families for my confrères, with the itch before the source was discovered.

There is probably not a gentleman in this audience that does even a small amount of negro practice in any of our cities who does not know of cases of syphilis in such a contagious stage from an extra-genital sense that they are liable to infect their employers. I am now treating two cases of syphilis resulting from labial infection in which there is no question in my mind but that the origin was from a servant.

I have seen quite a number of cases of gonorrheal infection in children from their nurses, in which absolute proof was obtained, and it is quite probable that many of our cases of fulminant ophthalmia might be traced to the same source.

A wife or child infected with tuberculosis, syphilis or gonorrhea is a terrible penalty to pay for our ignorance of the physical condition of our servants.

There is nothing to be gained by multiplying examples; the personal experience of every gentleman in this audience will furnish him with a sufficient number.

Our position is most peculiar. The servant comes to us professionally as a patient, and he has his absolute rights. We have no more moral or legal right to violate his professional confidence than we have to violate that of the highest person in the land. We can advise him of his condition and the dangers to which he is exposing others, and he can follow his own sweet will. We are legally powerless.

This is no time to discuss the race question. That the young negro of to-day, regardless of his emancipation, education and religion, is but a sad degenerate morally and physically in comparison with his ancestor of '61 is amply proven by that most interesting work of Hoffman, "Race Traits and Tendencies of the American Negro," as well as by the personal observation of the older members of this association.

That he is with us to stay is also proven by the efforts of colonization in Liberia and Kansas, as well as the most notable example of Durango. In fact, with all his increasing faults, he is as essential to us as we are to him, and will continue to be our servant for many generations.

In the present legal status of the question, there are two things that we can do. One is to encourage our servant to resume to a certain extent his former relations with us; to live in our houses and on our premises, and let us assume as far as possible under existing circumstances our old supervision of his physical condition and material welfare. This of course would entail great care and a large expense on our side. The other is to make a physician's certificate an essential to his employment, said certificate to be renewed from time to time. If we are willing to assume the expense of these examinations the vast majority of the applicants would not object. Thus far I have never had an objection from an applicant for the position of wet nurse, neither have I known any case of infection from an accepted one.

The applicant could be given to understand that the certificate would not divulge any of his physical ailments, but would simply state that he was or was not physically suitable for the prospective employment.

The danger to our children is much more serious than to us, because of the intimate personal contact between them and our servant. I am informed by high legal authority that owing to the peculiar legal status of our cities, occupying as they do a position between that of the state and the family, and especially the almost absolute power of the health authorities, an ordinance can be passed in any of them making it unlawful for any one afflicted with a known contagious disease to assume the care of a child. Also that the powers of the board of health in the various cities are practically limitless in regard to infected houses.

I bring this subject before the association, hoping for a full discussion; for instead of decreasing, these dangers are constantly on the increase, and from year to year, as we depart more and more from the simpler life of our ancestors, we are surrounded by new and ever-increasing dangers in our house and from our servant.

